# Managing chondral lesions of the glenohumeral joint

**DOI:** 10.4103/0973-6042.44143

**Published:** 2008

**Authors:** Richard S. Page

**Affiliations:** Orthopaedic Shoulder and Upper Limb Surgeon and Director of Barwon Orthopaedic Research Unit, Geelong Hospital, Victoria, Australia

Isolated chondral lesions of the glenohumeral joint, particularly in younger patients, are one of the management challenges of shoulder surgery. These lesions can result in shoulder pain and mechanical symptoms. They are often associated with other pathology, particularly to the labrum or biceps anchor, and may be associated with instability. These lesions can result from an impaction-type injury to the glenohumeral joint, such as the gleno-labral articular disruption lesion (GLAD) described by Naviaser in 1993. This injury was stated as being caused by forced adduction with the shoulder in abduction and external rotation.[1] Articular lesions can arise on the glenoid or the humeral surfaces, or on both. The natural history of these lesions has not been clearly defined, but when associated with instability or labral pathology, they are a precurser to osteoarthritis of the shoulder.

There is scant literature on the management of chondral lesions within the shoulder, particularly on attempts at chondral surface regeneration. The knee has received much more attention in this regard and algorithms for managing lesions within the shoulder have grown from an understanding of chondral pathology within the knee.

Treatment options initially relied on open surgery but more recently arthroscopic lavage, debridement, chondral abrasion, and drilling have been used. None of these procedures address the chondral defect and only address the unstable chondral surface. Attempts at chondral resurfacing have included periosteal transplant, microfracture arthroplasty, mosaicplasty, and chondral autologous grafting. Microfracture arthroplasty is a technique that some surgeons employ, but there are no reports in literature of it being used in isolation for the treatment of chondral lesions in the shoulder.

The Steadman technique of microfracture arthroplasty was published in 1999[2] with further outcomes published in 2001[3] and 2003. It should be noted that there is a meticulous and clearly outlined follow-up and rehabilitation protocol that this group adheres to in both the postoperative management and the follow-up of patients treated with this method.

With regard to the use of this technique in the shoulder there has been little published, and the paper by Snow on microfracture of chondral lesions of the glenohumeral joint in this edition of the IJSS aims to provide a little more data regarding outcomes.[4] Pässler, in the German literature in 2000, described the use of microfracture in the shoulder in discussing 162 patients who were followed up following microfracture arthroplasty of a range of joints and reported overall good results, particularly for pain relief at 3–6 years. However, it is not clear from an analysis of the results and levels of function how well shoulder lesions performed. Siebald *et al.* in 2003 reported cartilage repair in five patients with chondral defects using microfracture plus periosteal flap repair,[5] but it is not clear from their series what benefit was derived from the addition of the periosteal flap.

The paper in this issue of IJSS deals specifically with the detailed outcomes of microfracture in the shoulder. It should be noted that although this is a small series, it is a well-defined group. All lesions were full thickness and less than 4 cm^2^ in total surface area. In all but one patient a number of other procedures were performed at the same time. The results show that at a minimum of 15 months there was a significant improvement in the Constant score as well as the Oxford shoulder score. The outcomes in this small series are encouraging and certainly warrant further investigation and follow-up, ideally through a randomized controlled analysis of the outcomes with this method.

The treatment methodology involves specific instrumentation and is inexpensive compared to some other techniques, avoiding the expense and potentially uncertain long-term outcomes of chondrocyte grafting. The limitations of this technique include the technical aspects of the site of the lesions, as well as the difficulty in treating bipolar lesions. However, the arthroscopic application and retention of the sub-chondral plate, means that ‘no bridges are burnt’ if further treatment is required.

This series adds to the small amount of literature on this topic in the shoulder. To maximize the stability of the fibrocartilage as it matures it is necessary to minimize the shear forces on the joint surface during early healing of the ‘super clot’; consideration should, therefore, be given to transferring the postoperative rehabilitation concepts applied in the knee to the care of the shoulder also. In moving forward in our understanding of the management of chondral lesions, it will be important to standardize minimum reporting criteria of lesion geometry and additional procedures, as well as outcome measures, so that results may be compared. A true understanding of the mid- to long-term outcomes will ultimately require a multicenter, randomized controlled trial.
